# Synergistic effects of magnetic water treatment and mulching on crop and soil moisture-salinity distribution

**DOI:** 10.1038/s41598-025-98802-6

**Published:** 2025-05-06

**Authors:** Ahmed Abdelfattah, Montaser Awad, Omnia Sorour

**Affiliations:** Department of Agricultural and Biosystems Engineering, Faculty of Agriculture, Benha University, Moshtohor, Toukh, Qalyubia Egypt

**Keywords:** Brackish water, Magnetic water treatment, Mulching, Soil properties, Water productivity, Plant sciences, Environmental sciences, Natural hazards

## Abstract

Freshwater scarcity has increased the reliance on low quality water for irrigation purposes. Brackish and/or high salinity irrigation water may increase soil salinity and reduce yields. This study was carried out for two consecutive seasons to study the effect of magnetic treatment of brackish water and soil mulching on strawberry growth and productivity and soil moisture-salinity distribution. For this purpose, three irrigation water types were used: tap water (W_1_), brackish water (W_2_), and magnetically treated brackish water (W_3_). Four different soil mulches were evaluated: rice straw mulch applied at rate of 3 t ha^−1^ (M_1_), rice straw mulch applied at rate of 5 t ha^−1^ (M_2_), white polyethylene plastic mulch (M_3_), and black polyethylene plastic mulch (M_4_) compared to bare soil (M_0_). The results revealed that magnetic water treatment (MWT) and soil mulching significantly enhanced crop growth and productivity and improved soil moisture-salinity distribution. The difference between M_2_ and M_4_ was not statistically significant in almost all the studied traits in both growing seasons. This result highlights the potential of using rice straw as a sustainable alternative to plastic mulch in strawberry cultivation. Strawberry marketable yield and water productivity increased significantly by 26.7% and 18.6% over the two growing seasons as a result of MWT, compared to untreated water. Moreover, MWT had a positive effect on reducing soil salinization. MWT led to a significant decrease in soil salinity by 17.8% compared to untreated water (W_2_) and the difference in soil salinity between W_1_ and W_3_ was not statistically significant. The integration of MWT and straw mulch at 5 t ha^−1^ (W_3_M_2_) has resulted in marketable yield increase of 32.6 and 40.9% compared to brackish water irrigation and bare soil conditions (W_2_M_0_). Hence W_3_M_2_ could be adopted as a sustainable management practice for safe use of brackish irrigation water in strawberry cultivation

## Introduction

The challenge of limited water availability is one of the most critical issues in modern agriculture. As global population grows and food demands increase, the competition for freshwater resources has intensified. This has resulted in a pressing need for the development and implementation of innovative and sustainable water management strategies. Traditional freshwater sources are progressively being depleted or contaminated, making it imperative to explore alternative water sources for irrigation. It is estimated that more than 40% of the global population is affected by water scarcity. Approximately 2.2 billion people worldwide lack access to freshwater resources^1,2^.

Egypt faces severe water scarcity crisis. Egypt’s dependence on the Nile makes it particularly vulnerable to fluctuations in water availability. The Nile’s flow is influenced by rainfall patterns in the upstream countries and by infrastructure developments like dams. Additionally, rapid urbanization and industrial activities contribute to the overuse and pollution of existing water resources. Climate change exacerbates these issues, potentially altering rainfall patterns and reducing water share. As of recent estimates, Egypt’s per capita water availability is dangerously low and projected to decline further. The per capita water share have radically declined from 2526 m_3_/year in 1947 to less than 700 m^3^/year in 2013, and projections indicate that it may fall to less than 350 m^3^/year by 2050^3,4^.

Given the pressing need for sustainable water management, utilizing brackish water for irrigation could present a promising solution. Although there is no precise definition for brackish water, it is generally recognized as surface or groundwater with salinity level higher than freshwater and lower than seawater (between 1000 and 10,000 ppm). Water may be categorized according to its salinity level into three main classes: (1) seawater with salinity level of ≥ 35,000 ppm, (2) brackish water or medium-salinity water with salinity level of 1000:15,000 ppm, and (3) fresh water with salinity level of ≤ 500 ppm, also known as low-salinity water^5^.

Brackish water is not suitable for direct consumption but can be adapted for agricultural use with appropriate technology. High salinity levels can lead to reduced soil fertility, impaired plant growth, and decreased crop yields. Salinity adversely affects the osmotic balance in plants, making it difficult to absorb water and essential nutrients. It is reported that continuous irrigation with brackish water has restricted crop growth and resulted in yield reductions by 32–46%^6,7^. Therefore, effective treatment of brackish water is crucial to mitigate these adverse effects and ensure sustainable agricultural productivity.

Two promising approaches may address the challenges associated with irrigation with brackish water, magnetic water treatment (MWT) and the use of soil mulch. The process of MWT involves passing water through a magnetic field to alter its physical and chemical properties, potentially improving its interaction with soil and plants^8^. MWT has been shown to reduce the viscosity and surface tension of water, which could increase its permeability, thereby facilitating water and nutrient uptake by plant roots. MWT can disrupt hydrogen bonding between water molecules. This could improve soil moisture dynamics, enhance water uptake by plants, and increase crop yields^9,10^. MTW has been reported to reduce the contact angle of water on smooth surfaces and increase its ability to penetrate micropores. Enhanced water movement may help leach salts from the root zone, reducing the overall soil salinity^11,12^.

Soil mulch, on the other hand, helps conserve soil moisture, regulate temperature, and reduce evaporation. These benefits are particularly important when using high salinity irrigation water. They may help mitigate the negative effects of salt accumulation and maintain optimal soil conditions for plant growth^8,13^.The use of plastic mulches in agriculture has substantially increased over the past decade. Plastic mulching has proven its efficiency in enhancing crop yields, conserving soil moisture, and suppressing weed growth. Despite the fact that these benefits are significant, notable limitations associated with the use of plastics in agriculture must be considered. Plastics are derived from fossil fuels and contribute to greenhouse gas emissions during production. Additionally, disposing of consumed plastics may cause serious environmental pollution. They break down over time and release toxic chemicals that pose a severe threat to the ecosystem. Furthermore, the physical presence of plastic waste in soil can hinder water infiltration, root growth, and ultimately affect crop productivity^14,15^. In response to these limitations, there is a growing interest in exploring biodegradable alternatives made from natural materials such as rice straw and other organic compounds. These alternatives aim to provide similar benefits without the long-term environmental consequences associated with traditional plastic mulches.

Strawberry (*Fragaria x ananassa*) is a widely cultivated crop for its economic value and nutritional benefits. The global demand for strawberry continues to rise. Egypt has been ranked fourth globally in strawberry production after China, the United States (US) and Turkey, with a production of 597,029 tons^16^. Al-Deir region in Kalubia governorate represents the highest share in strawberry production and accounts for 70% of the total strawberry production in Egypt. Although the area dedicated to strawberry cultivation in this region increased by 8.3% over the past two years, productivity per hectare decreased by 28%. The growth and yield of strawberry can be significantly affected by saline conditions of both irrigation water and soil. Most strawberry growers in Al-Deir region depend on groundwater as the main source of irrigation water. However, there has been an increase in the salinity of both irrigation water and soil in the last decade, necessitating the need for drilling new wells or accepting significant reductions in yield and profitability. Alternatively, effective management practices may enhance crop growth and productivity under high salinity conditions.

Despite the potential benefits of MWT and soil mulching, there is limited research on their combined effects on soil and crop water productivity specifically in salt-sensitive crops like strawberry. This research aims to evaluate the interactive effects of MWT and soil mulches on soil moisture and salinity distribution and strawberry crop performance, thereby providing insights for optimizing brackish water use in agriculture. The study also aims at evaluating rice straw as sustainable and biodegradable alternative to plastic mulches in strawberry cultivation.

## Materials and methods

### Experimental site

**Fig. 1:**
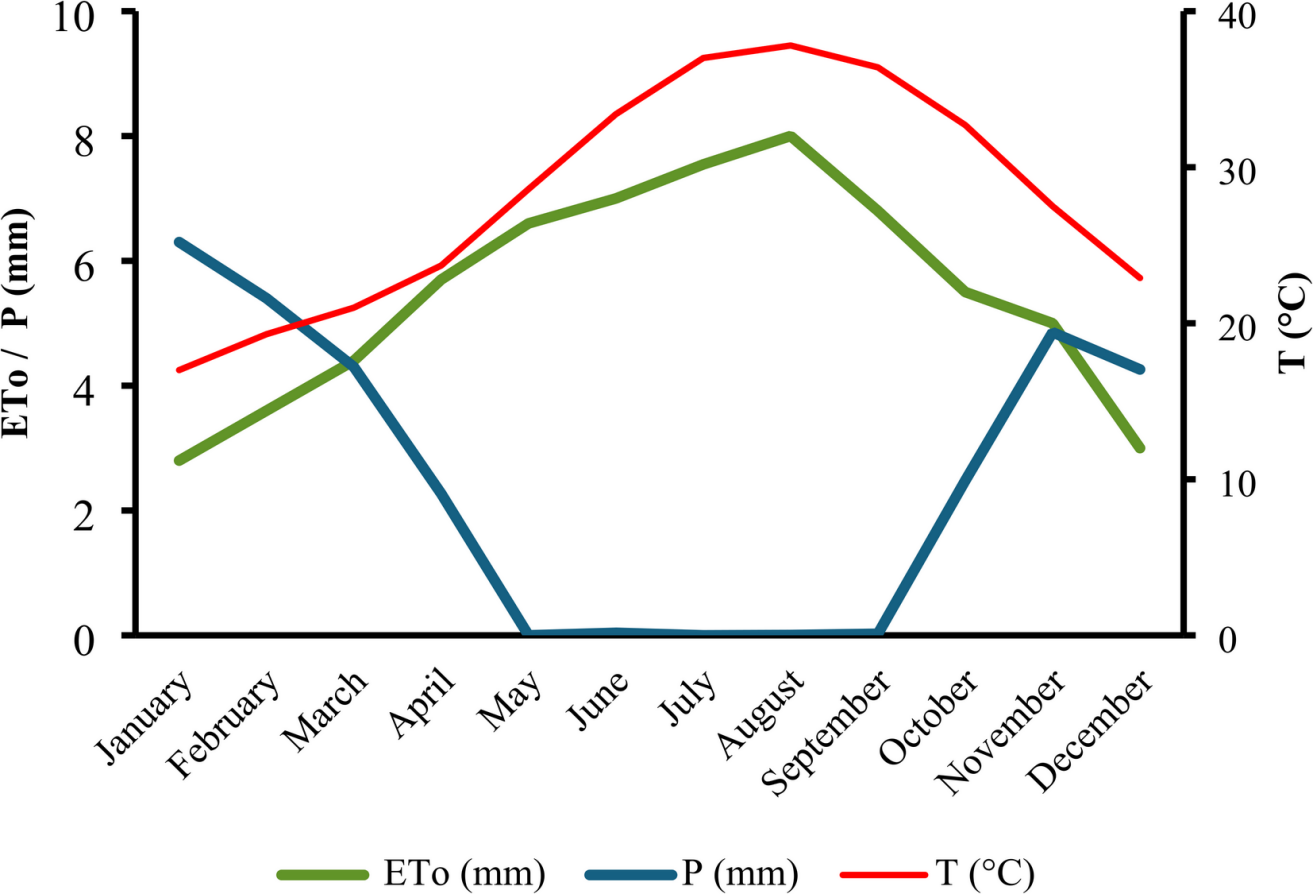
Average values of monthly meteorological data during 2023 and 2024 in the experimental site, where, T, maximum temperature (°C), ETo, reference evapotranspiration (mm) and P, effective precipitation (mm).

Field experiments were conducted on September 15th during two successive growing seasons, 2023 and 2024. The experiment was located in Al-Deir, Toukh City, Kalubia Governorate, Egypt (30° 20′ N, 31° 16′ E). The area is characterized by an arid dry climate, with annual precipitation of less than 37 mm. The average values of daily weather parameters are shown in Fig. 1. The soil texture of the experimental site was loam, and the chemical and physical properties of the soil are shown in Tables 1 and 2.

**Table 1:** Physical and hydro-physical soil properties.

Particle size distribution					
Soil depth (cm)	Sand (%)	Silt (%)	Clay (%)	Textural class	
0–20	50.55	33.45	16.00	Loamy	
20–40	51.00	34.50	14.50		
40–60	49.80	34.60	15.60		

Hydro-physical properties					
Depth (cm)	FC (%)	WP (%)	AW (%)	K (cm h^−1^)	BD (g cm^−3^)
0–20	24.50	13.11	11.39	1.36	1.40
20–40	24.30	13.22	11.08	1.31	1.43
40–60	25.73	10.33	15.40	1. 25	1.46

*FC, Field capacity; WP, Wilting point, AW, Available water, K, Hydraulic conductivity, BD, Bulk density.

**Table 2:** Chemical properties of soil.

	pH	E.C (dS/m)	Soluble cations (ppm)				soluble anions (ppm)			
			Ca^++^	K^+^	Na^+^	Mg^++^	Cl^−^	SO_4_^=^	HCO_3_^−^	CO_3_^=^
0–20	7.60	1.54	3.29	1.64	5.78	4.66	5.75	2.72	6.89	–
20–40	7.54	1.66	3.98	1.78	6.37	4.22	6.42	3.68	6.25	–
40–60	7.29	1.75	4.76	2.13	6.74	3.84	6.95	4.78	5.74	–

### Experimental design

**Fig. 2:**
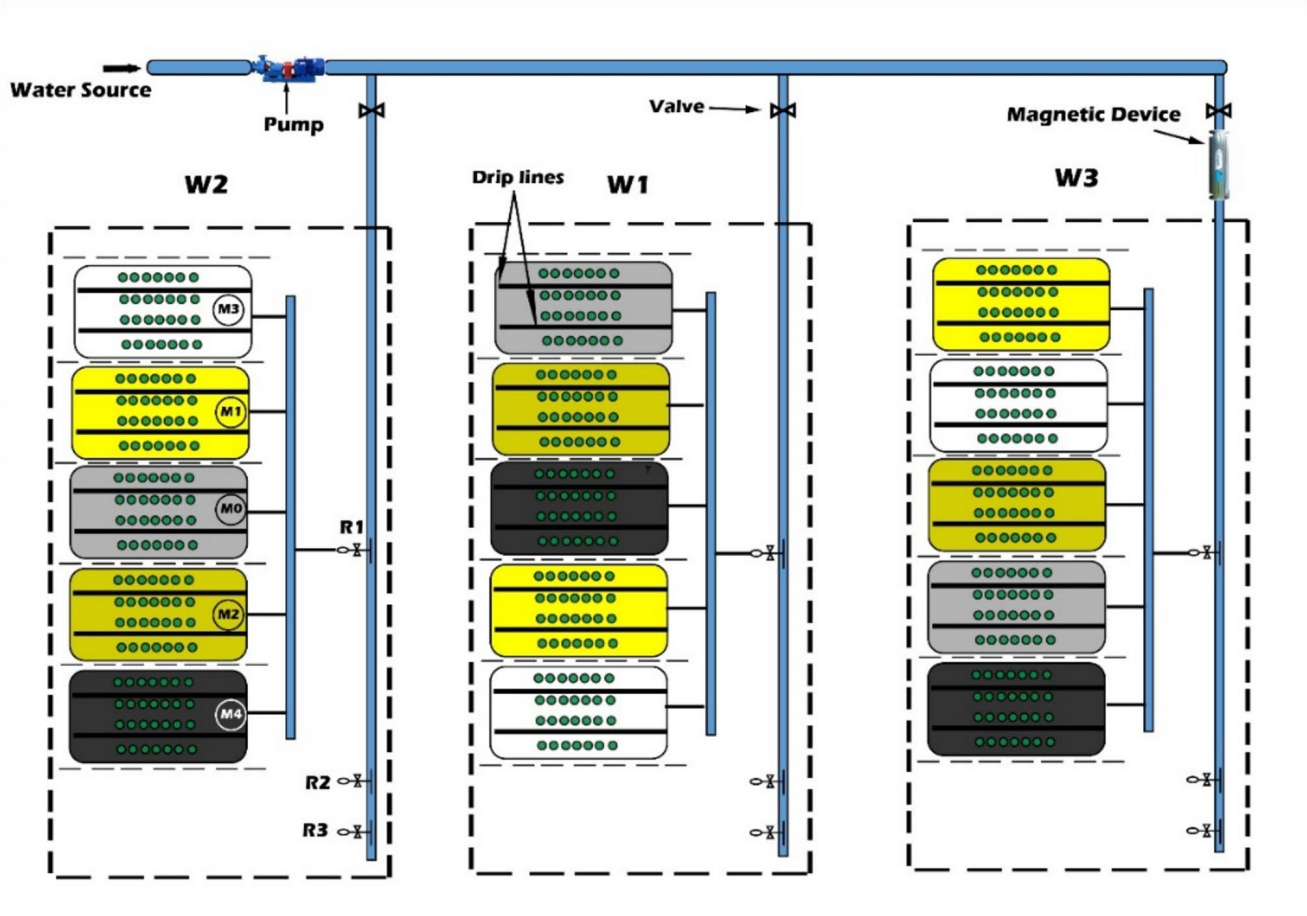
Schematic of the experimental set up and field layout.

To evaluate the potential of MWT and soil mulching on strawberry growth and productivity and soil moisture-salinity distribution, a factorial experiment was carried out. The experimental design was applied using a split plot layout in a randomized block design with three replicates for each treatment. Irrigation water type was assigned as the main plot factor and included three levels: tap water (W_1_), untreated well water (W_2_), and magnetically treated well water (W_3_). Four different soil mulches were assigned to the subplots: bare soil (M0), rice straw mulch applied at rate of 3 t ha^−1^ (M1), rice straw mulch applied at rate of 5 t ha^−1^ (M2), white polyethylene plastic mulch (M3), and black polyethylene plastic mulch (M4). Seedlings were transplanted on raised beds of 30 m length, 120 cm width and 40 cm height. The distance between plants was 25 cm, and 30 cm between rows (four plant rows/bed). Each treatment consisted of three beds and was replicated three times. The layout of the experiment is shown in Fig. 2.

### Transplantation and irrigation management

Seedlings of strawberry (*Fragaria x ananassa* cv. Festival.) were transplanted on 15th of September of each growing season. Seedlings were obtained from Strawberry and Non-Traditional Crops Development Center, Faculty of Agriculture, Ain Shams University, Egypt. Plants were irrigated using drip irrigation system equipped with built-in emitters having a discharge rate of 4 l/h. Two lateral lines of 16 mm diameter were used for each bed. A 5 m^3^ tank was used to supply water for W_1_ treatments, while the main source for irrigation water in W_2_ and W_3_ was groundwater well having salinity level of 1200 ppm (1.9 dS/m). Chemical characteristics of irrigation water is presented in Table 3.

**Table 3:** Chemical analysis of irrigation water.

	PH	EC dS/m	EC ppm	Soluble cations (ppm)				Soluble anions (ppm)			
				Ca^++^	K^+^	Na^+^	Mg^++^	Cl^−^	SO_4_^−^	HCO_3_^−^	CO_3_^−^
W_1_	7.3	0.45	288	1.75	1.08	0.47	1.22	1.64	0.75	2.2	–
W_2_	7.6	1.9	1216	4.22	2.24	6.87	5.84	6.64	5.65	6.79	–

* W_1_, Tap water and untreated well water W_2_.

The water magnetization process was carried out using a magnetic source device produced by Delta water Co. for water treatment, Alexandria, Egypt. The device operates by utilizing a strong magnetic field (14,500 Gauss or 1.45 Tesla) to alter the physical structure of water without changing its chemical composition. The device has a length of 85 cm and an inner diameter of 2 inches (5.08 cm). To minimize magnetic interference and enhance water magnetism, the device is composed of inner magnets and protected by a stainless-steel shield. The device is installed around the water pipe so that magnets are oriented in such a way that they attract each other, which generates a concentrated magnetic field within the pipe^17^.

Irrigation water requirements were determined by measuring soil moisture content on a volumetric basis. Soil moisture content was continuously monitored using the VH400 dielectric soil moisture sensor and VG-METER-200 hand-held moisture meter (Vegetronix, Inc., Riverton, Utah, USA). The sensor is based on capacitive measurement principle and generates an alternating electric field around its electrodes that penetrate the surrounding soil. The higher the volumetric soil moisture content, the higher is the permittivity (dielectric constant) of the soil and therefore the resulting total capacitance of the probe. Irrigation was scheduled in all treatments when 50% of the available soil moisture within the effective root depth was depleted^18^.

Irrigation water requirements $I$ (m^3^ ha^−1^) was calculated according to^19^ as follows:

$$I=\frac{\left({\theta }_{fc}-{\theta }_{i}\right)}{100}\times {D}_{r}\times P\times 10$$

where ${\theta }_{fc}$: volumetric soil moisture content at field capacity (%), ${\theta }_{i}$: volumetric soil moisture content before irrigation (%), ${D}_{r}$: the effective root depth (mm), and $P$: the amount of allowable depletion or the readily available water (for strawberry, P was assumed 50% of the total available water in the soil).

Soil mulching was performed 40 days after transplanting. Standard black and white polyethylene mulches of 50-micron thickness and 180 cm width were applied. Rice straw mulching was applied at rates of 3 and 5 t ha^−1^. All experimental units and replicates were subjected to the same agricultural practices in terms of irrigation levels, fertilization, and pest and disease control.

### Plant measured parameters

At each harvest fully red berries were picked, and yield parameters i.e. yield (g/plant) and total yield (kg/ha) were recorded. At the end of each growing season ten plants were randomly selected from each treatment and the following vegetative growth parameters were recorded: plant height (cm), number of leaves per plant and chlorophyll content. Chlorophyll content was measured using portable nondestructive chlorophyll meter, SPAD-502 (Konica Minolta Inc., Tokyo, Japan).

Water Productivity (WP) of strawberry under different treatments was calculated. WP refers to the effectiveness with which plants convert water into biomass or yield. It can be quantified by measuring the ratio of the fruit yield to the volume of water consumed during the growing season. The WP (kg m^−3^) of strawberry was calculated according to the following Eq. ^20^:

$$WP=\frac{Y}{{ET}_{c}}$$

where $Y$: total yield (Kg ha^−1^), ET_c_: seasonal water consumption (m^3^ ha^−1^).

### Soil measurements

Soil moisture content and salinity for different treatments were evaluated. Periodic soil sampling was carried out using a 5 cm diameter auger. Soil samples were taken at 20 cm intervals from 0 to 60 cm and soil moisture contents were evaluated on the basis of oven dry weight. The soil samples were immediately weighed, and oven dried at 105 °C for 24 h or until constant weight was reached. Gravimetric (${\theta }_{m})$ and volumetric (${\theta }_{v}$) soil moisture contents were calculated according to the following Eqs. ^21^:

$${\theta }_{m}= \frac{{W}_{1}-{W}_{2}}{{W}_{2}} \times 100$$

$${\theta }_{v}={\theta }_{m}\times {\rho }_{b}$$

where ${W}_{1}$ is weight of wet soil (g), ${W}_{2}$ is weight of dry soil (g) and ${\rho }_{b}$ is soil bulk density.

Volumetric soil moisture (${\theta }_{v}$) content was subsequently expressed as profile moisture content in the 0–60 cm soil depth. At the end of the growing season, the salt content of soil samples was evaluated using 1:5 soil–water extract^22^. The electrical conductivity (EC) of the soil extract was measured using a calibrated portable digital EC meter (Jenway 4510 Conductivity/TDS Meter; 230 VAC/UK).

### Statistical analysis

The obtained results of both growing seasons were statistically analyzed using Statistix software package (version 10, Tallahassee FL, USA). Data were subjected to analysis of variance (ANOVA) and means were compared using the least significant difference (LSD test at 5% confidence level).

## Results

### Effect of different treatments on crop growth parameters

The data in Table 4 show the effect of irrigation water type and soil mulch on some growth characteristics of strawberry plants. Based on the results, it is clear that all measured growth characters were negatively affected by brackish water treatment (W_2_) compared to tap water (W_1_) and treated water treatments (W_3_). The lowest number of leaves, plant height and chlorophyll content was achieved under W_2_ in both growing seasons. The data revealed that magnetic treatment of brackish water (W_3_) significantly increased the number of leaves, plant height and chlorophyll content compared to untreated water (W_2_) by 21.6, 10.4 and 18.6%, respectively, in 2023 and by 17.3, 14.1 and 18.1%, respectively, in 2024. Furthermore, no significant differences were observed between W_1_ and W_3_ in most of the characteristics studied, Table 4.

**Table 4:** Effect of water treatments and type of soil mulch on strawberry growth parameters.

Soil mulch type	2023			2024		
	Water treatments					
	W1	W2	W3	W1	W2	W3
No. of leaves per plant						
M0	21.3^e^	15.7^f^	20.7^e^	21.7^efg^	17.7^h^	23.0^de^
M1	24.0^cd^	17.7^f^	21.7^e^	23.0^de^	21.0^g^	22.7^def^
M2	26.0^abc^	20.3^e^	25.0^bc^	26.3^abc^	23.3^d^	26.7^ab^
M3	25.7^abc^	21.0^e^	24.3^bcd^	25.7^bc^	21.3^fg^	25.0^c^
M4	27.7^a^	22.3^de^	26.3^ab^	27.3^a^	22.7^def^	27.0^ab^
LSD at 0.05	1.9			1.4		
Plant height (cm)						
M0	22.3^ef^	19.0^g^	22.0^ef^	22.0^efg^	18.6^h^	22.0^efg^
M1	24.0^de^	21.0^fg^	24.0^de^	23.7^d−g^	21.0^gh^	24.0^c−f^
M2	25.7^bcd^	23.6^de^	27.3^ab^	27.7^ab^	24.3^cde^	28.0^a^
M3	25.0^cd^	21.3^f^	26.7^abc^	25.0^bcd^	21.3^fgh^	26.7^abc^
M4	28.7^a^	22.3^ef^	27.3^ab^	28.7^a^	23.3^d−g^	27.7^ab^
LSD at 0.05	1.5			1.6		
Chlorophyll content						
M0	47.7^ef^	42.3^h^	48.3^def^	49.3^b−e^	40.3^g^	48.3^def^
M1	50.0^b−e^	44.7^gh^	49.0^c−f^	50.0^b−e^	44.0^fg^	49.0^cde^
M2	52.0^ab^	48.0^ef^	52.3^ab^	53.7^ab^	47.7^ef^	52.7^a−d^
M3	51.3^abc^	46.7^fg^	52.0^ab^	53.7^ab^	46.7^ef^	53.0^abc^
M4	52.7^a^	47.0^fg^	50.7^a−d^	56.0^a^	48.3^def^	56.0^a^
LSD at 0.05	2.5			4.8		

Means followed by the same letter (s) within each row, column or interaction are not significantly different at 5% level.

The results also showed that soil mulching significantly affected strawberry growth parameters (*P* ˂ 0.05). The lowest significant values of the measured vegetative characteristics were achieved under bare soil (M_0_). Moreover, the number of leaves, plant height and chlorophyll content significantly increased under M_4_ (black polyethylene mulch) by 32.4, 8.7 and 23.7%, respectively, in 2023 and by 23.5, 16.2 and 27.1%, respectively, in 2024 as compared to the control (M_0_). These parameters also increased significantly under M_2_ (straw mulch applied at 5 t ha^−1^) by 23.7, 10.1 and 21.1%, respectively, in 2023 and by 22.5, 11.6 and 27.7%, respectively, in 2024 as compared to the respective control (M_0_). Furthermore, the difference between M_2_ and M_4_ was not statistically significant in almost all the studied traits in both growing seasons (Table 4)**.**

### Effect of different treatments on soil moisture distribution

**Fig. 3:**
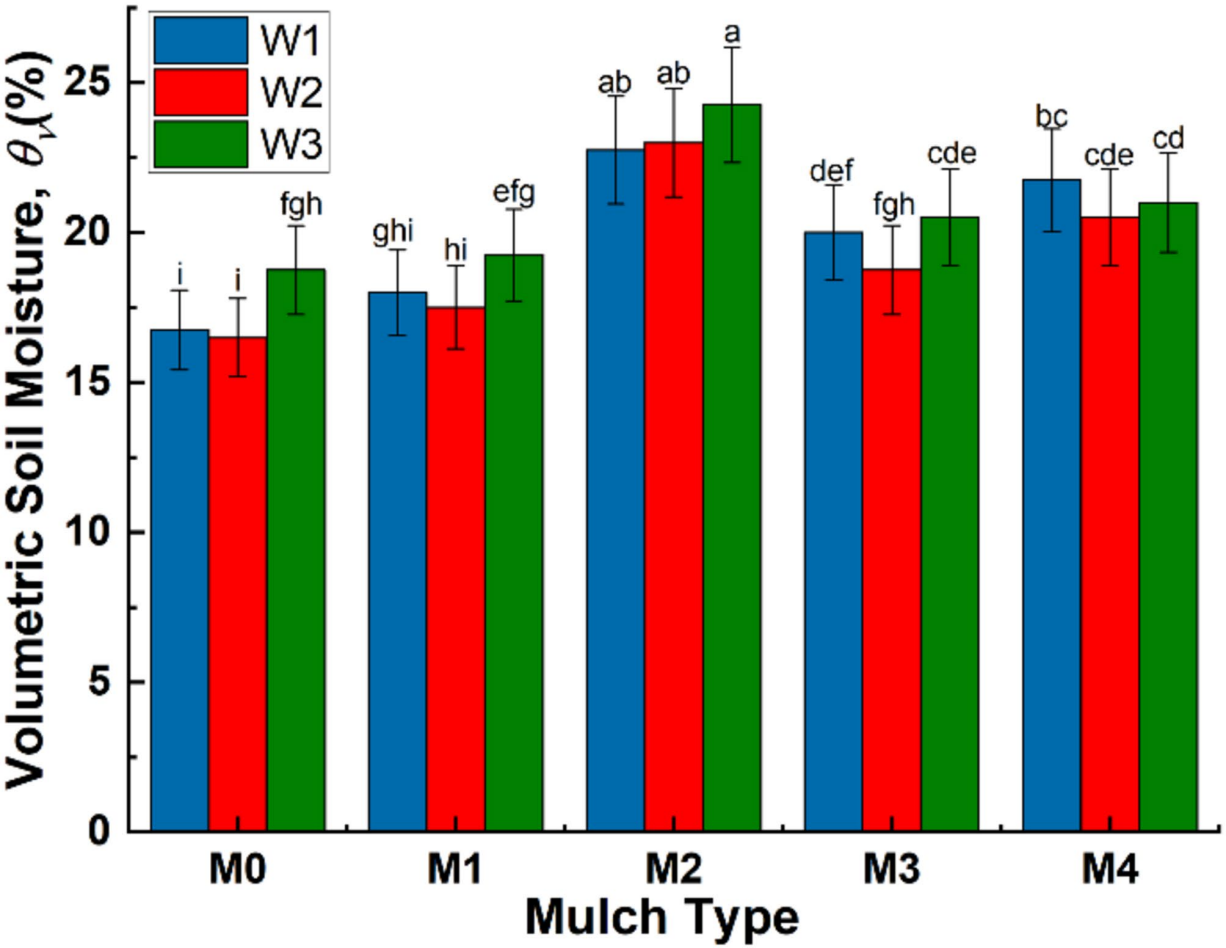
Average volumetric moisture in soil profile depth of (0–60 cm).

**Fig. 4:**
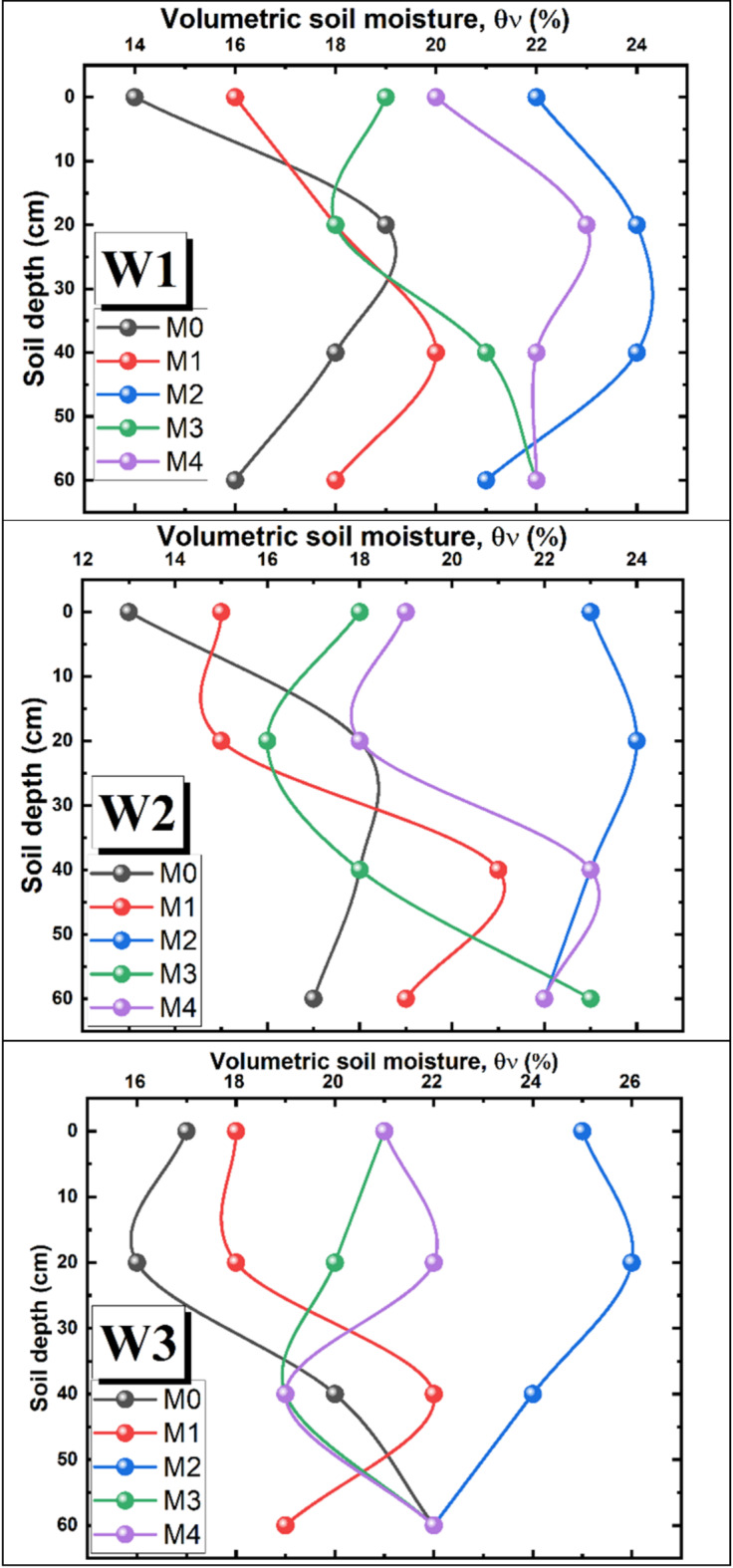
Soil moisture distribution under different treatments.

Figures 3 and 4 show the combined effect of irrigation water and mulch materials on soil moisture. The results indicated a significant difference in volumetric soil moisture contents (${\theta }_{v}$) depending on both soil mulch type and irrigation water. The highest ${\theta }_{v}$ values were recorded under W_3_ (magnetically treated water), followed by W_1_ and W_2_, respectively. However, the difference was not statistically significant between W_1_ and W_2_. The magnetic treatment of W_3_ has led to an increase in ${\theta }_{v}$ by 4.5 and 7.8% as compared to tap water (W_1_) and untreated brackish water (W_2_), respectively.

Regarding the effect of mulch type on ${\theta }_{v}$, it was observed that the highest significant ${\theta }_{v}$ values were achieved under M_2_ followed by M_4_, M_3_ and M_1_, respectively. While the lowest ${\theta }_{v}$ values were recorded under the control (bare soil, M_0_). Utilizing straw mulching at M_2_ application rate has resulted in increased ${\theta }_{v}$ by 34.7%, as compared to the control. The increase in ${\theta }_{v}$ under M_2_ was 10.7% and 18.1% compared to black and white polyethylene mulching, respectively. In addition, the white (M_3_) and black (M_4_) polyethylene mulching has led to increased ${\theta }_{v}$ values by 14.5 and 21.7%, respectively, as compared to the control. There was no significant difference in ${\theta }_{v}$ values between bare soil (M_0_) and low application rate of straw mulching, M_1_.

The maximum ${\theta }_{v}$ value of soil profile was 26% and was recorded under W_3_M_2_ at 20 cm depth, while the lowest value was 13% and was recorded under W_2_M_0_ at soil surface, Fig. 4. Moreover, the results showed that regardless of irrigation water type, straw mulching at M_2_ application rate caused an increase in ${\theta }_{v}$ values at the soil surface compared to the depth of 60 cm, which may be indicative of a decrease in evaporation from the soil surface. Straw mulch acts as a barrier that limits direct exposure of the soil surface to sunlight and temperature and this insulation of soil surface helps to maintain a more stable temperature regime. At high temperatures, the soil does not heat up as quickly, which further reduces evaporation rates. For tap water W_1_, ${\theta }_{v}$ values were lower at soil surface compared to ${\theta }_{v}$ at 60 cm depth by 12.5, 11, 13.6 and 9% under M_0_, M_1_, M_3_ and M_4_, respectively. While for M_2_, ${\theta }_{v}$ values were higher at soil surface by 4.8% compared to 60 cm soil depth. A similar trend was observed under W_2_ and W_3_.

### Soil salinity distribution under different treatments on

**Fig. 5:**
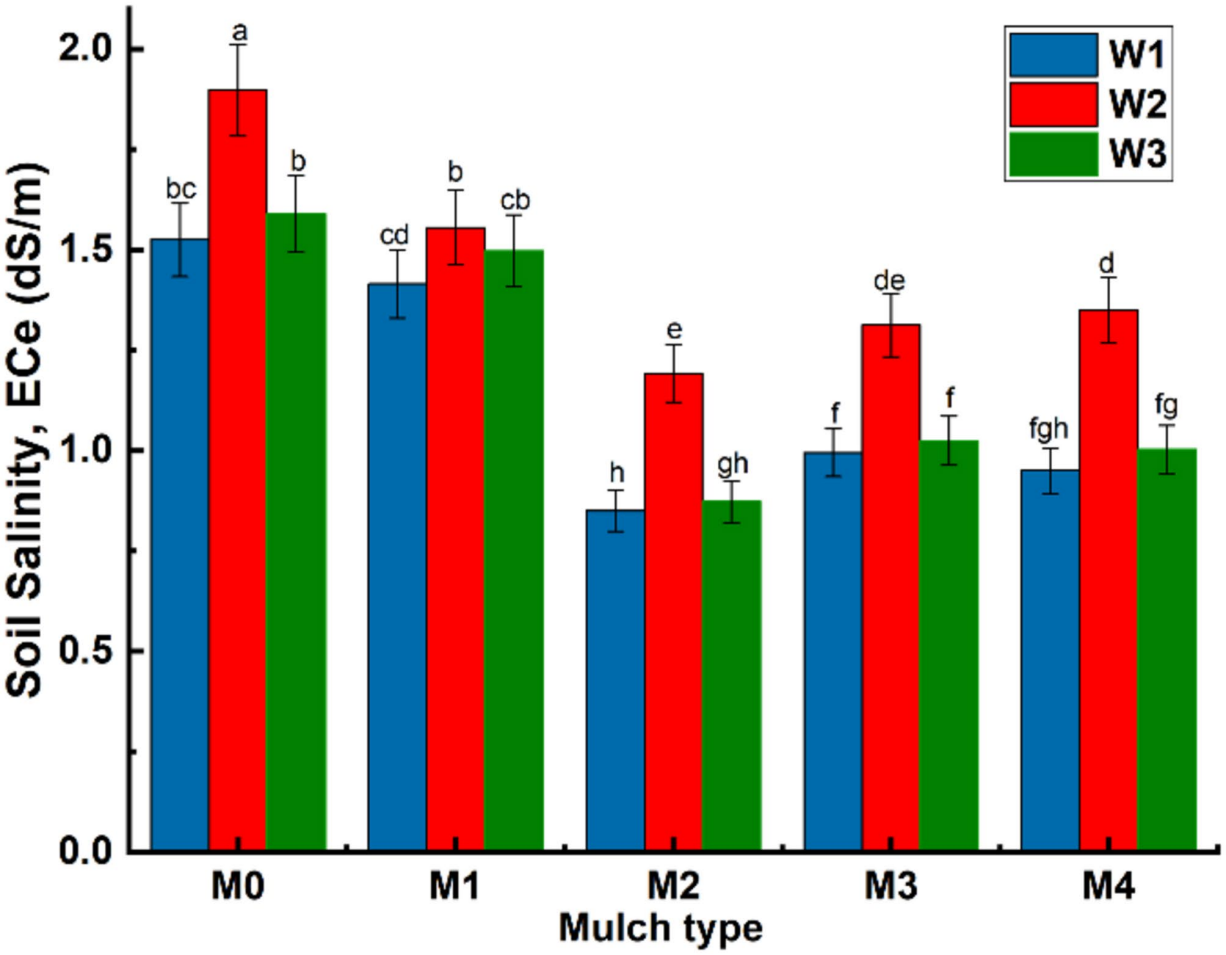
Average salinity of soil profile under different soil mulches and water treatments.

**Fig. 6:**
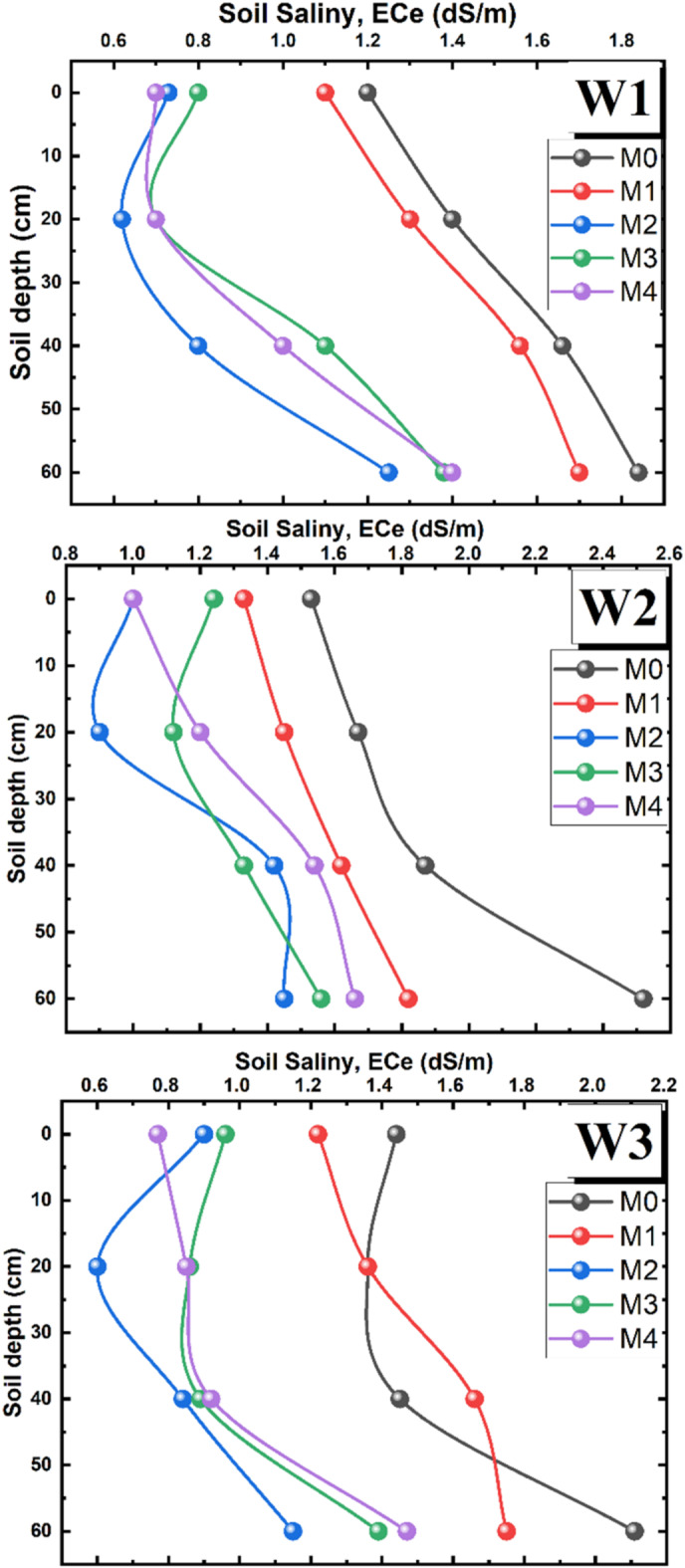
Soil profile salinity distribution under different treatments.

The effect of different treatments on soil salinity EC (dS/m) is shown in Figs. 5 and 6. The results showed that EC (dS/m) differed significantly under different levels of water and mulch. The highest EC (dS/m) values was recorded under W_2_ (untreated brackish water), followed by W_3_ and W_2_, respectively. Magnetic water treatment had a positive effect on reducing soil salinization, as the difference in soil EC values between W_1_ and W3 was not statistically significant. The magnetic treatment of W_3_ has led to decreased of soil EC values by 17.8% as compared to untreated water (W_2_).

The results also revealed that for all water types, a significant decrease in soil salinity was recorded under the mulched soil treatments compared to the control (non-mulched treatments M_0_). It was also noticed that the lowest salt accumulation in the 0–60 cm soil depth was achieved under M_2_ (organic soil mulch), followed by M_4_, M_3_ and M_1_, respectively. The utilization of M_2_ has resulted in soil salinity decrease of 42% as compared to the control. In addition, the white (M_3_) and black (M_4_) polyethylene mulching has led to decreased EC values by 34%, as compared to the control. The difference in EC values (dS/m) between white and black polyethylene mulch was not statistically significant.

It was also observed that EC values (dS/m) of bare soil treatments (M_0_) gradually increased with depth until it reached its highest value at 60 cm depth, under all water types. In contrast, a decrease in soil salinity values was observed at surface depths from 0 to 20 cm under all mulching treatments. This indicates that mulching acts as a barrier that reduces evaporation from the soil surface and thereby increases moisture retention. Furthermore, the maximum EC value of soil profile was 2.52 (dS/m) and was recorded under W_2_M_0_ at 60 cm depth, while the lowest value was 0.6 (dS/m) and was recorded under W_3_M_2_ at 20 cm depth, Fig. 6.

### Effect of different treatments on strawberry yield

The data in Table 5 represent the effect of soil mulch cover and water type on strawberry crop productivity. The results indicate that irrigation water quality had a more significant impact on productivity than soil mulch cover. The results showed that irrigation water type significantly affected strawberry marketable yield (*P* ˂ 0.05%). For both growing seasons, the highest marketable yield (g/plant) was achieved under tap water (W_1_) followed by W_3_ (magnetized water) and W_2_ (untreated brackish water), respectively. However, the difference between W_1_ and W_3_ was not statistically significant. These results demonstrate that the MWT process enhances strawberry production under brackish irrigation water conditions. Marketable yield increased significantly by an average of 26.7% over the two growing seasons as a result of MWT, compared to untreated water.

**Table 5:** Effect of water and soil mulch types on strawberry yield and WP.

Soil mulch type	2023			2024		
	Water treatments					
	W1	W2	W3	W1	W2	W3
Yield (g/plant)						
M0	550.6^c^	457.7^ef^	555.3^c^	551.7^d^	435.6^g^	561.3^d^
M1	587.3^b^	447.6^f^	574.0^b^	589.0^b^	464.3^f^	575.3^c^
M2	611.7^a^	477.0^d^	607.0^a^	611.7^a^	476.3^e^	614.0^a^
M3	607.3^a^	466.0^de^	589.7^b^	606.7^a^	465.3^f^	591.0^b^
M4	612.7^a^	476.3^d^	608.3^a^	615.3^a^	481.0^e^	611.7^a^
LSD at 0.05	17.1			9.6		
WP (kg/m^3^)						
M0	9.12^d^	8.63^ef^	9.40^d^	9.04^e^	8.37^g^	9.31^d^
M1	9.81^c^	8.44^f^	9.85^c^	9.94^c^	8.88^ef^	9.78^c^
M2	10.51^b^	8.79^e^	10.51^b^	10.46^b^	8.69^f^	10.77^a^
M3	10.52^b^	8.53^ef^	10.40^b^	10.45^b^	8.47^ g^	10.39^b^
M4	10.60^ab^	8.64^ef^	10.87^a^	10.90^a^	8.73^f^	10.93^a^
LSD at 0.05	0.31			0.18		

Means followed by the same letter (s) within each row, column or interaction are not significantly different at 5% level.

Concerning the effect of soil mulch, the results showed that mulching positively affected marketable yield in both seasons. Marketable yield showed a significant increase for all soil mulch covers compared to the control (*P* < 0.05%). The highest yield (g/plant) was achieved under M_4_ followed by M_2_, M_3_ and M_1_, respectively, While the lowest value was recorded under the bare soil treatment M_0_. Marketable yield increased significantly by 8.5 and 10.3% for 2023 and 2024, respectively, under M_4_, compared to M_0_ (bare soil treatments). Similarly, yield significantly increased by 8.4 and 10% for 2023 and 2024, respectively, under M_2_ as compared to M_0_.

Although the highest yield was achieved under M_4_ (black polythene mulch), the difference between M_4_ and M_2_ (rice straw mulch applied at 5 t ha^−1^) was not statistically significant. This result highlights the potential of using rice straw as a sustainable alternative to both black and white polythene plastic mulch in strawberry cultivation.

With regard to the effect of interaction, the highest marketable yield was achieved under W_1_M_4_ for both growing seasons, followed by W_1_M_2_ in 2023 and W_3_M_2_ in 2024. On the other hand, the lowest marketable yield was recorded under W_2_M_0_ (brackish water and bare soil) and W_2_M_1_ (brackish water and straw mulch applied at 3 t ha^−1^). Moreover, the integration of MWT and straw mulch at 5 t ha^−1^ (W_3_M_2_) has resulted in marketable yield increase of 32.6 and 40.9% compared to brackish water irrigation and bare soil conditions (W_2_M_0_). Hence W_3_M_2_ could be adopted as a sustainable management practice for safe use of brackish irrigation water in strawberry cultivation.

### Effect of different treatments on strawberry water productivity

The effect of different treatments on water productivity (WP) is shown in Table 5. The results indicated that irrigation water significantly affected strawberry WP (*P* ˂ 0.05%). The highest WP (kg m^−3^) was achieved under W_3_ followed by W_1_ and W_2_, respectively, in both seasons. The average values of 2023 and 2024 revealed that MWT enhanced WP by 18.6% compared to brackish water. The results also revealed that WP exhibited significant differences under all soil mulch covers. Compared to bare soil (M_0_), straw mulch at M_2_ has led to significant increase in WP by 9.8 and 12%, for 2023 and 2024, respectively. Whereas WP increased by 11% in 2023 and 14.3% in 2024 under M_4_ compared to M_0_.

Moreover, the interaction effect of irrigation water and soil mulch cover showed statistically significant variation for WP. For both growing seasons, the highest WP was recorded under W_3_M_4_ and was 10.87 and 10.93 kg m^−3^ for 2023 and 2024, respectively. On the other hand, the lowest WP was recorded under W_2_M_1_ and W_2_M_0_ for 2023 and 2024, respectively. Overall, the integration of MWT and soil mulch covers has resulted in an average increase in WP by 28.3, 25.3, 22.4, and 15.6% for W_3_M_4_, W_3_M_2_, and W_3_M_3_, and W_3_M_1_ respectively, compared to brackish water irrigation and bare soil conditions (W_2_M_0_).

## Discussion

Traditional freshwater sources are becoming increasingly scarce due to over-extraction and climate change impacts. The utilization of high salinity water for irrigation purposes may be necessary in water-limited regions. However, the high salt content of irrigation water can pose challenges to plant health and soil quality^23,24^.

Strawberry (Fragaria x ananassa) is considered one of the most commercially valuable crops in the horticultural sector. However, strawberry is characterized as salt-sensitive crop and can experience substantial yield reductions in salt-affected conditions. The results of this study revealed that irrigating strawberry with brackish water significantly reduced vegetative growth, yield, and water productivity. These results are consistent with^25,26^ who stated that salt stress conditions can potentially reduce the yield of strawberry by 33:50%.

The sensitivity of strawberry to salt stress can be attributed to their susceptibility to ion toxicity, oxidative and osmotic stress. High salinity induces the production of reactive oxygen species, causing oxidative damage. Salt stress may also lead to the accumulation of sodium in strawberry tissues, which can interfere with potassium uptake and cause ionic imbalance^27^. Furthermore, when plants are exposed to high salinity levels of both soil and irrigation water, the salt concentration outside the root cells exceeds that inside, creating an osmotic imbalance that draws water out of the plant cells. As a result, plants experience soil water deficit, which impairs their uptake of water and essential nutrients. This can ultimately lead to wilting, stunted crop growth, and substantial yield loss if salt stress is not mitigated^28,29^. Sustainable management practices such as magnetic water treatment (MWT) and soil mulching can mitigate adverse effects of salt stress.

The data revealed that MWT significantly enhanced strawberry vegetative growth, yield and water productivity. MWT significantly increased the number of leaves, plant height and chlorophyll content compared to brackish untreated water. Furthermore, no significant differences were observed between tap and MWT in most of the characteristics studied. The marketable yield increased significantly by an average of 26.7% over the two growing seasons as a result of MWT, compared to untreated water. Several studies suggest that MWT might alter the physical and chemical properties of water, such as reducing its surface tension. This reduction could increase its permeability, potentially facilitating more efficient uptake of water and nutrients by plant roots^30,31^. It is also reported that MWT promotes soil aggregation, which creates larger pore spaces that improve both aeration and moisture retention. This can eventually alleviate the osmotic stress caused by high salt concentrations, ensuring that plant roots can effectively access soil moisture^32,33^. Water molecules are held together by hydrogen bonds and tend to form clusters. It is suggested that when water is exposed to magnetic fields, the alignment of water molecules is altered. MWT may disrupt hydrogen bonds, reducing the size of water clusters. This could improve soil moisture dynamics, enhance water uptake by plants, and potentially increase crop yields^34–36^. Our results confirmed that MWT increased volumetric soil moisture content and reduced salinity levels.

The results also indicated a significant increase in crop growth parameters and marketable yield for all soil mulch covers compared to bare soil. The enhanced crop productivity under soil mulch covers can be attributed to improved soil moisture distribution, which lowers the concentration of soluble salts and reduce salinity levels. Additionally, soil mulching resulted in significantly higher moisture content compared to bare soil treatments. By reducing evaporation, soil mulch covers help retain soil moisture, which is particularly crucial in saline conditions where high evaporation rates can concentrate salts in the crop root zone^37^.

Furthermore, there was no significant difference in marketable yield between polyethylene and straw mulch applied at the rate of 5 t h^−1^. This result highlights the potential of using rice straw as a sustainable alternative to both black and white polythene plastic mulch in strawberry cultivation. Straw mulch is biodegradable and contributes to soil health as it decomposes, enriching the soil with organic matter and nutrients. This improves soil structure, supports a diverse range of beneficial microorganisms, and enhances nutrient cycling, ultimately improving overall soil health^38,39^. Furthermore, straw is often more cost-effective, especially for small-scale farms. In contrast, plastic polyethylene mulch is non-biodegradable, leading to long-term environmental pollution as it breaks down into microplastics that can harm soil microorganisms and ecosystems. Additionally, plastic mulch can create anaerobic conditions by restricting air exchange, which reduces oxygen availability for soil organisms and plant roots^15,40^.

Many studies have highlighted the potential of MWT to enhance crop growth and productivity. However, there are also limitations that must be carefully considered. Variations in magnetic field strength may influence crop response differently. A magnetic field of 1800–2000 G was used to treat the water^32^, while it was 3860 Gauss in the study by putti et al.^41^. This highlights the need for standardized experimental protocols to validate the observed effects under diverse conditions. Although the short-term benefits of MWT are encouraging, the long-term effects on soil structure, microbial communities, and environmental sustainability require further investigation.

Overall, the results demonstrated that the integration of MWT and soil mulching led to significant improvements in crop growth, yield, and water productivity (WP). It also provided optimal conditions for crop growth, characterized by higher soil moisture contents and reduced salt concentration, compared to irrigation with non-magnetized water and bare soil conditions.

## Conclusion

Water scarcity is a growing challenge worldwide, necessitating the use of brackish and low-quality water for irrigation purposes. Innovative approaches to ensure sustainability and mitigate the risks associated with salinity hazards are therefore crucial. The results indicated that both MWT and soil mulching significantly enhanced crop growth and productivity and resulted in lower soil salinity compared to brackish irrigation water and bare soil conditions. The results also revealed that the integration of magnetic water treatment and soil mulching may represent a synergistic approach to enhance agricultural productivity and reduce soil salinity. The integration of MWT and straw mulch at 5 t ha^−1^ (W_3_M_2_) has resulted in marketable yield increase of 32.6 and 40.9% compared to brackish water and bare soil conditions (W_2_M_0_). Furthermore, the difference between straw mulching applied at 5 t ha^−1^ and plastic mulch covers was not statistically significant in almost all the studied traits in both growing seasons. This result highlights the potential of using rice straw as a sustainable alternative to plastic mulch in strawberry cultivation. Similarly, the difference between tap and MTW was not statistically significant in most of the studied attributes. Long term studies are required, especially under higher levels of irrigation water salinity and crops with varying salinity tolerance.

## Data Availability

The datasets used and/or analyzed during the current study are available from the corresponding author on reasonable request.
